# Lipid rafts mediate multilineage differentiation of human dental pulp-derived stem cells (DPSCs)

**DOI:** 10.3389/fcell.2023.1274462

**Published:** 2023-11-09

**Authors:** Francesca Santilli, Jessica Fabrizi, Stefano Martellucci, Costantino Santacroce, Egidio Iorio, Maria Elena Pisanu, Mattea Chirico, Loreto Lancia, Fanny Pulcini, Valeria Manganelli, Maurizio Sorice, Simona Delle Monache, Vincenzo Mattei

**Affiliations:** ^1^ Biomedicine and Advanced Technologies Rieti Center, “Sabina Universitas”, Rieti, Italy; ^2^ Department of Experimental Medicine, “Sapienza” University of Rome, Rome, Italy; ^3^ High Resolution NMR Unit, Core Facilities, Istituto Superiore di Sanità, Rome, Italy; ^4^ Department of Biotechnological and Applied Clinical Sciences, University of L’Aquila, L’Aquila, Italy; ^5^ Dipartimento di Scienze della Vita, della Salute e delle Professioni Sanitarie, Link Campus University, Rome, Italy

**Keywords:** DPSCs, lipid rafts, gangliosides, osteogenic, chondrogenic and adipogenic differentiation, multilineage differentiation, mesenchymal stem cells, dental pulp stem cells

## Abstract

Cell outer membranes contain glycosphingolipids and protein receptors, which are integrated into glycoprotein domains, known as lipid rafts, which are involved in a variety of cellular processes, including receptor-mediated signal transduction and cellular differentiation process. In this study, we analyzed the lipidic composition of human Dental Pulp-Derived Stem Cells (DPSCs), and the role of lipid rafts during the multilineage differentiation process. The relative quantification of lipid metabolites in the organic fraction of DPSCs, performed by Nuclear Magnetic Resonance (NMR) spectroscopy, showed that mono-unsaturated fatty acids (MUFAs) were the most representative species in the total pool of acyl chains, compared to polyunsatured fatty acids (PUFAs). In addition, the stimulation of DPSCs with different culture media induces a multilineage differentiation process, determining changes in the gangliosides pattern. To understand the functional role of lipid rafts during multilineage differentiation, DPSCs were pretreated with a typical lipid raft affecting agent (MβCD). Subsequently, DPSCs were inducted to differentiate into osteoblast, chondroblast and adipoblast cells with specific media. We observed that raft-affecting agent MβCD prevented AKT activation and the expression of lineage-specific mRNA such as OSX, PPARγ2, and SOX9 during multilineage differentiation. Moreover, this compound significantly prevented the tri-lineage differentiation induced by specific stimuli, indicating that lipid raft integrity is essential for DPSCs differentiation. These results suggest that lipid rafts alteration may affect the signaling pathway activated, preventing multilineage differentiation.

## 1 Introduction

Dental pulp is a rich and accessible source of multipotent stem cells called Dental pulp-derived stem cells (DPSCs) with mesenchymal stem cells (MSCs) characteristics and the ability to differentiate into cells of several mesodermal tissues, including cartilage, bone, skeletal and cardiac muscles (Delle Monache et al., 2021; Delle Monache et al., 2022). DPSCs show the same *in vitro* MSCs’ properties (Aydin and Sahin, 2019; Xiao et al., 2021) and exhibit plasticity, high proliferative ability, self-renewal, and multilineage differentiation capability (Mattei et al., 2015; Delle Monache et al., 2022). MSCs (including DPSCs) produce Exosomes, containing cytokines and miRNAs, strategically released into target tissues, creating bidirectional interactions between cells and the environment, and play a vital role in stem cell therapy (Codispoti et al., 2018; Tatullo et al., 2019). Numerous studies (Mattei et al., 2015; Tsutsui, 2020; Xiao et al., 2021) have shown the capability of DPSCs, under specific culture conditions, to differentiate into several cell cytotypes such as odontoblasts, osteoblasts, neural cells, chondrocytes, adipocytes, myoblasts, fibroblasts and pericytes (Liu et al., 2015; Nuti et al., 2016; Martellucci et al., 2018; Martellucci et al., 2019; Staniowski et al., 2021). Recently, it has been discovered that several cellular components, i.e., glycosphingolipids, are involved in the induction of DPSCs’ early neuronal differentiation (Mattei et al., 2015; Santilli et al., 2022). Beyond their roles as structural components of cells, lipids on plasma membrane, including sphingolipids, ceramide and cholesterol, function as bioactive molecules in many important complex biological processes, ranging from cell division to the multilineage differentiation. Within each lipid class, cells produce a variety of individual lipid species (i.e., carbon chain length, desaturation, and hydroxylation variants), whose importance is only beginning to be investigated (Nguyen et al., 2017). For instance, short-chained and saturated lipids generate a more tightly packed cell membrane, whereas on the other, longer-chained, and unsaturated lipids are more mobile in membranes, thereby increasing fluidity and providing greater opportunities for lipids and proteins to interact. In particular, the production of monounsaturated fatty acids (MUFAs) is catalyzed by stearoyl-CoA desaturases (SCDs), a family of membrane-bound non-heme iron-containing enzymes. The preferred substrates are palmitoyl- and stearoyl-CoA, which are then converted into palmitoleoyl-and oleoyl-CoA, respectively (Paton and Ntambi, 2009). These products are the most abundant MUFAs and serve as substrates for the synthesis of various kinds of lipids, including gangliosides (Ntambi and Miyazaki, 2004; Paton and Ntambi, 2009), mainly enriched in membrane microdomains in which the preferential associations between cholesterol and saturated lipids drive the formation of relatively packed (or ordered) membrane domains that selectively recruit certain lipids and proteins, named *lipid rafts* (Martellucci et al., 2020; Santilli et al., 2022). Lipid rafts are small, heterogeneous, and highly dynamic lipid domains in the cell membranes and fall into two broad categories, non-caveolar lipid rafts and caveolae, based on the absence or presence of caveolin proteins, respectively (Sohn et al., 2018). Lipid rafts are enriched in cholesterol, sphingolipids, and proteins, forming platforms that function in membrane signaling and trafficking (Iwabuchi, 2018; Martellucci et al., 2020; Mattei et al., 2020; Li L et al., 2022). Lipid rafts can dissociate and associate rapidly, forming functional clusters in cell membranes (Kraft, 2017) and are distributed on the membrane of sub-cellular organelles, including Endoplasmic Reticulum (ER), Golgi apparatus, endosomes, lysosomes, and lipid droplets but also in mitochondria and nuclei (Cascianelli et al., 2008; Hayashi and Fujimoto, 2010; Sorice et al., 2012a; Sorice et al., 2012b; Manganelli et al., 2015; Tsai et al., 2016; Wang et al., 2020). Based on the biochemical nature, lipid rafts act as platforms for cellular and/or exogenous proteins (Barbat et al., 2007) with different functions including the shuttling of molecules on the cell surface, the organization of cell signal transduction pathways, the entry of pathogens, and the conversion of cellular prion protein (PrP^C^) in the pathological isoform (Bieberich, 2018; Azzaz et al., 2022) named scrapie prion protein (PrP^Sc^) (Martellucci et al., 2020). Furthermore, lipid rafts play a pivotal role in regulating a variety of signal transduction pathways responsible for specific cellular programs, including apoptosis, proliferation, differentiation, stress responses, necrosis, inflammation, autophagy, and senescence, thus determining cell fate (Sorice et al., 2010; Sezgin et al., 2015). In addition, it has been demonstrated that lipid rafts play an important role in endocytosis process of many viruses and promote the entry of bacterial pathogens into non-phagocytic cells (Chen et al., 2019). Moreover, lipid rafts may control the release of microvesicles including exosomes and this process is compromised after lipid rafts’ perturbation (Feng et al., 2020). Several studies revealed that lipid rafts are involved in life cycle of different microorganisms and viruses, including coronaviruses (Fecchi et al., 2020), such as in the severe acute respiratory syndrome by Coronavirus-2 (SARS-CoV-2), the causal agent of Coronavirus Disease 2019 (COVID-19) (Li et al., 2021; Palacios-Rápalo et al., 2021). Gangliosides (GGs) are the main sphingolipids present in lipid rafts (Hannun and Obeid, 2018; Li B et al., 2022) and involved in several cellular processes (Codini et al., 2021). GGs such as GM3, GM1, and GD3 (Martellucci et al., 2020; Codini et al., 2021), are glycosphingolipids (GSLs) consisting of ceramide and a bulky sugar chain that contains one or more sialic acids (Sarmento et al., 2020). They are ubiquitously distributed in tissues and body fluids and highly expressed in various regions of the central nervous system (CNS) (Schnaar et al., 2014; Sarbu et al., 2022). GGs are important biological molecules involved in various physiological processes including cell proliferation, adhesion, migration, apoptosis, cell-cell interactions, cell differentiation (Ryu et al., 2009; Yang et al., 2011; Moussavou et al., 2013; Ryu et al., 2020) and interact with proteins such as the epidermal growth factor receptor (EGF-R), involved in signal transduction (Bieberich, 2018; Kim et al., 2021). It has been shown that GGs play an important role both in mouse embryonic stem cells and in DPSCs’ neuronal differentiation process (Lee et al., 2007; Ryu et al., 2009). GGs are also strongly correlated with brain disorders through aberrant glycosylation pathways (Ledeen and Wu, 2018) and an excess of GGs or the disruption of lipid rafts (Grassi et al., 2020) seems to cause severe neurodegenerative disorders (Sarbu et al., 2022), like Huntington’s disease (HD), Alzheimer’s disease (AD), Parkinson’s disease (PD), amyotrophic lateral sclerosis (ALS), stroke, multiple sclerosis (MS) and epilepsy (Alpaugh et al., 2017; Dukhinova et al., 2019; Magistretti et al., 2019; Zhang et al., 2019; Malinick et al., 2020; Sipione et al., 2020; Bouscary et al., 2021; Yahi et al., 2021). The GGs could also play an important role in DPSCs’ differentiation (Lee et al., 2010; Moussavou et al., 2013; Ryu et al., 2020). In fact, our previous studies have shown how lipid rafts are involved in DPSCs’ neuronal differentiation process (Mattei et al., 2015; Martellucci et al., 2018; Martellucci et al., 2019). For this reason, this work is focused on the identification and quantification of lipid species in DPSCs, the analysis of expression variability in GGs composition in different lineage-committed cells, with the aim to investigate if lipid rafts can play a significant role in DPSCs’ multilineage differentiation processes.

## 2 Materials and methods

### 2.1 Cell culture

DPSCs were purchased from Lonza (Walkersville, United States) and cultured in Dental Pulp Stem Cell BulletKit™ Medium which includes both basal medium and the necessary supplements for human dental pulp mesenchymal stem cell proliferation (Lonza, Walkersville, United States), in a humified incubator under the atmosphere of 5% CO_2_ at 37°C. The culture medium was replaced every 3 days and when 90% confluence was achieved, cells were harvested using 0.05% Trypsin-EDTA (Euroclone, Milan, Italy). Cells were cultured between P4-P8 for subsequent experiments, and each was repeated at least three times.

### 2.2 Treatments for DPSCs’ multilineage differentiation

To induce DPSCs’ multilineage differentiation process we use the following protocol: DPSCs were seeded at the density of 2 × 10^4^ cells/well in 6-well culture dishes (Sarstedt, Milan, Italy) with basal growth medium. After over-night attachment, the basal medium was replaced with specific culture differentiation media to induce Osteogenic, Chondrogenic, and Adipogenic differentiation prepared as shown in Table 1. Every 3 days the differentiation media were replaced with fresh complete differentiation media.

**TABLE 1:** Reagents, concentrations, and time used for the 3-differentiation media preparation.

*Reagents*	*Osteogenic Differentiation Medium (ODM)*	*Chondrogenic Differentiation Medium (CDM)*	*Adipogenic Differentiation Medium (ADM)*
DMEM-LG	1X	1X	1X
FBS	15%	—	10%
Penicillin/Streptomycin	100 U/mL	1%	—
L-ascorbic acid phosphate	0.1 mM	50 μg/mL	—
Dexamethasone	0.01 μM	100 nM	1 μM
Beta-glycerophosphate	0.01 mM	—	—
L-Glutamine	2 mM	—	—
TGF-β1	—	10 ng/mL	—
Sodium pyruvate	—	1 mM	—
ITS	—	50 mg/mL	—
BSA	—	1.25 mg/mL	—
Insulin	—	—	10 μg/mL
Indomethacin	—	—	200 μM
3-Isobutyl-1-methylxanthine (IBMX)	—	—	0.5 mM
Time of stimulation for AKT Phosphorylation	10 min		
Time of stimulation for Differentiation	14 days	21 days	21 days
Time of stimulation for Real-Time PCR	48 h		

### 2.3 Ganglioside’s extraction and HPTLC analysis

All samples obtained as reported above were subjected to ganglioside extraction according to Svennerholm and Fredman (Svennerholm and Fredman, 1980). Briefly, the samples were extracted twice in chloroform/methanol/water (4:8:3) (v/v/v) and subjected to Folch partition by the addition of water resulting in a final chloroform/methanol/water ratio of 1:2:1.4. The upper phase, containing polar glycosphingolipids, was purified of salts and low molecular weight contaminants using Bond elut C18 columns (Superchrom, 07807T, Restek Corporation, Bellefonte, PA), according to the method of Williams and McCluer (Williams and McCluer, 1980). The eluted glycosphingolipids were dried and separated by high-performance TLC (HPTLC) (Yu and Ariga, 2000), using silica gel 60 HPTLC plates (Merck, Sigma-Aldrich, Milan, Italy). Chromatography was performed in chloro-form/methanol/0.25% aqueous KCl (5:4:1 v/v/v). Plates were then air-dried, and gangliosides visualized with resorcinol.

### 2.4 Lipidic profile of DPSCs by nuclear magnetic resonance (NMR) spectroscopy

For the extraction of lipid metabolites, cell pellets were extracted according to the protocol previously described (Saulle et al., 2021). The dried samples were resuspended in 750 μL of solution CDCl3:CD3OD (2:1; v/v), containing 0.02% tetramethylsilane (TMS) for chemical shift for NMR analyses. High-resolution 1H-NMR analyses were performed at 25°C at 600 MHz (14.1 T Bruker AVANCE Neo spectrometer; Karlsruhe, Germany, Europe) on organic cell extracts using standard 1D Bruker library 1H-NMR spectra. A total of 512 scans were collected into 32,768 data points using a spectral width of 12,500 Hz, an acquisition time of 1.31 s and a relaxation delay (d1) of 5 s. Prior to Fourier transformation, each FID (free induction decay) was zero-filled to 65,536 points and multiplied by a 0.3 Hz exponential line-broadening function. Subsequently, spectra were manually phased, baseline corrected, and chemical shifts referenced internally to TMS at d = 0.00 ppm by using Topspin software 4.1. Relative quantification (area) of lipid signals in organic fractions was normalized to number of cells. The chemical shift of characteristic lipid signals was reported in Supplementary Table S1.

### 2.5 Lipid raft perturbation

The perturbation of the lipid rafts can be induced using different approaches by targeting specific substrates. As lipid raft perturbation molecules, we used methyl-β-cyclodextrin (MβCD) since this compound is known to induce cholesterol and sphingolipids release from the membrane (Ottico et al., 2003; Mahammad and Parmryd, 2015; Abe and Kobayashi, 2021). The cells were pretreated with MβCD (Merck, Sigma-Aldrich, Milan, Italy) 5 mM for 30 min at 37°C in 5% CO_2_ before stimulation with three differentiation specific media as specified above. Preliminary experiments demonstrated that cell viability after MβCD treatment was about 95%.

### 2.6 Western blot analysis

DPSCs, untreated or treated with MβCD for 30 min and stimulated with specific three differentiation media for 10 min, as described above, were subjected to sodium dodecyl sulfate-polyacrilamide gel electrophoresis (SDS-PAGE). Briefly, DPSCs were lysed in lysis buffer containing 0.1% Triton X-100 (Merk, Sigma Aldrich, Milan, Italy), 10 mM Tris-HCl (pH 7.5), 150 mM NaCl, 5 mM EDTA, 1 mM Na_3_VO_4_ and 75 U of aprotinin and allowed to stand for 20 min at 4°C. The cell suspension was mechanically disrupted by Dounce homogenization (10 strokes). The lysates were centrifuged for 5 min at 1,300 × g to eliminate nuclei and large cellular debris and, after protein concentration analysis by Bradford Dye Reagent assay kit (Bio-Rad, Milan, Italy), the lysates were tested with 10% sodium dodecyl sulfate-polyacrylamide gel electrophoresis (SDS-PAGE). Subsequently, the proteins were electrophoretically transferred to PVDF membranes (Life Technologies, Monza, Italy) with iBLOT2 and blocked with 5% bovine serum albumin (BSA) (Euroclone, Milan, Italy) in TBS containing 0.05% Tween 20 (Bio-Rad, Milan, Italy), and probed with rabbit anti-AKT pAb and rabbit anti-total AKT pAb (Cell Signaling Technology Danvers, MA, United States). Antibodies were visualized with horseradish peroxidase (HRP)-conjugated anti-rabbit IgG (Cell Signaling Technology, Danvers, MA, United States) and immunoreactivity assessed by chemiluminescence reaction, using the ECL detection system (Thermo Scientific, Rockford, United States) with myECL Imager (Life Technologies, Monza, Italy). Densitometric scanning analysis were accomplished with NIH Image 1.62 software by Mac OS X (Apple Computer International).

### 2.7 Reverse transcription-quantitative PCR (RT-qPCR) analysis

DPSCs untreated or treated with differentiative media (Table 1) in presence or not of MβCD were analyzed by RT-qPCR analysis. DPSCs were cultured in 6-well culture dishes (Euroclone, Milan, Italy), 1,25 × 10^5^ cells per well; at the end of the treatments, the total cellular RNA was extracted using TRIzol^®^ Reagent (Thermo Fisher Scientific, Rockford, IL, United States), and its quality and quantity were evaluated on a NanoDrop spectrophotometer (Thermo Fisher Scientific, Rockford, IL, United States). For RT-qPCR analysis we used specific human primers, sense, and antisense (Table 2). For the Real-Time amplification, the Luna Universal qPCR One-step kit (BioLabs, New England) was used according to the instructions, which allows amplifying the various genes starting directly from the RNA. In addition, the amplification of the genes of interest was done, and in parallel, that of the housekeeping gene glyceraldehyde-3-phosphate dehydrogenase (GAPDH) was performed as a positive reference. The amplification reaction was performed on a MiniOpticon Real-Time PCR System (Bio-Rad, Milan, Italy) using the following program: the RT reaction was set at an initial reverse transcription step at 55°C for 10 min, denaturation step at 95°C for 1 min, 40 amplification cycles at 95°C for 10 s, and 30 s at 60°C and melt curve at 60°C–95°C for 15 s. The relative expressions of the genes investigated were calculated using the comparative quantification method 2^−ΔΔCt^, with GAPDH serving as the reference gene.

**TABLE 2:** Primer sequences used in RT-qPCR analysis.

*Gene*	Forward primer	Reverse primer	*Tm Value* (°C)
GAPDH	CTG​CAC​CAC​CAA​CTG​CTT​AG	ACC​TGG​TGG​TCA​GTG​TAG​CC	60
OSX	ACG​GGT​CAG​GTA​GAG​TGA​GC	GGG​ATC​CCC​CTA​ATC​AAG​AG	60
SOX9	AGC​GAA​CGC​ACA​TCA​AGA​C	GCT​GTA​GTG​TGG​GAG​GTT​GAA	58
PPARγ2	GAA​CGA​CCA​AGT​AAC​TCT​CC	CGC​AGG​CTC​TTT​AGA​AAC​TCC	58.5

### 2.8 Staining techniques

#### 2.8.1 Alizarin red staining

DPSCs were fixed in formaldehyde (Euroclone, Milan, Italy) for 30 min. After this time, Alizarin Red S staining solution (Merck, Sigma-Aldrich, Milan, Italy) was added to cover the cellular monolayer and incubate at room temperature (RT) in the dark for 45 min. Carefully aspirate the Alizarin Red S staining solution and wash the cell monolayer four times with 1 mL of distilled water. Carefully aspirate the washing buffer and add phosphate buffered saline (PBS) (Euroclone, Milan, Italy).

#### 2.8.2 Oil red O staining

Oil red O solution (Merck, Sigma-Aldrich, Milan, Italy) was used for lipid droplet staining. Briefly, cells were fixed in 4% formalin/PBS for 30 min at RT. After fixation, the cells were washed in 60% isopropanol for 5 min. The isopropanol was aspirated completely, and the cells were incubated in 60% oil red O (0.3% in isopropanol solution) for 10 min at 37°C. The adipogenic differentiation was high-lighted by the lipid droplet accumulation stain red.

#### 2.8.3 Alcian blue staining

Alcian Blue solution (Merck, Sigma-Aldrich, Milan, Italy) was used to indicate synthesis of proteoglycans by chondrocytes. Briefly, cells were treated with 3% ascorbic acid (Merck, Sigma-Aldrich, Milan, Italy) at RT and were washed twice in PBS and incubated in 1% Alcian Blue-HCl 0.1 N for 30 min. Wells were rinsed twice with 0.1 N HCl and then were washed with distilled water before microscopic visualization and capturing. All images of each sample were visualized and captured using AxioVert. A1 inverted optical microscope (Carl Zeiss, Jena, Germany) through AxioVision^®^4.1 software (Carl Zeiss, Jena, Germany).

### 2.9 Statistical analysis

Quantitative analysis of immunoblot images and HPTLC analysis were carried out using NIH ImageJ as software (National Institutes of Health, United States). Statistical procedures were performed by GraphPad Prism software Inc. (San Diego, CA, United States). D’Agostino-Pearson omnibus normality test was used to assess the normal distribution of the data. Normally distributed variables were summarized using the mean ± standard deviation (SD). Differences between numerical variables were tested using Paired *t*-test. *p < 0.05, **p < 0.005 ***p < 0.001, ****p < 0.0001.

## 3 Results

### 3.1 Evaluation of basal lipidic content of DPSCs by NMR spectroscopy

**FIGURE 1:**
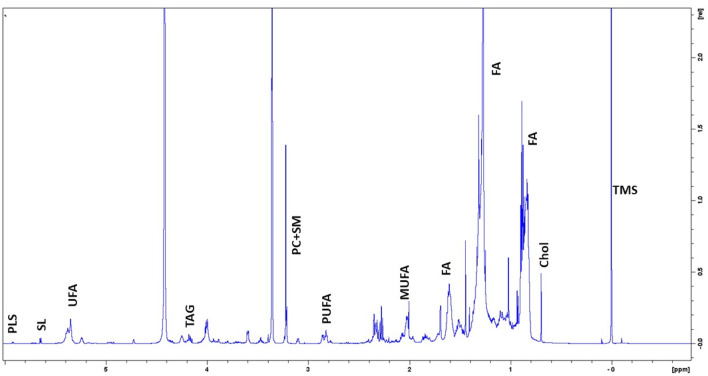
Representative 1H-NMR spectra of lipidome profile of DPSC cells. Peak assignment in organic fraction: signals of the pool of acyl chains (FA) determined at 0.9 ppm (ω-CH3 of FA), at 1.56 ppm (-CH2-CH2-COO of FA;) and at 2.40 ppm (-CH2-COO of FA); total cholesterol (Chol); monounsaturated fat acids (MUFA), plasmalogens (PLS), polyunsaturated FA (PUFA); sphyngolipids, (SL) unsaturated FA (UFA) pool of phosphatidylcholine (PC) plus Lyso-PC and Triacylglicerids (TAG); tetramethylsilane (TMS) as chemical shift internal reference.

To clarify the basal lipidome profile, 1H-NMR spectroscopy was performed in organic fraction of DPSC cells. Since the biophysical properties of lipids are tightly depended by the number of double bonds in the acyl chain, we first determined the ratio of mono-unsaturated Fatty Acids (MUFAs) to polyunsatured Fatty Acids (PUFAs) was 2.25 in organic extracts of DPSC cells, suggesting that MUFAs were the most representative species as compared PUFAs (Figure 1; Table 3).

**TABLE 3:** Relative quantification (Signal area normalized to number of cells) of lipid metabolites involved in the major pathways detectable by NMR spectroscopy (14.1T) in organic fraction of DPSC cells (*n* = 2).

Lipid metabolite	Mean ± half maximum deviation (n = 2)
plasmalogens	17.79 ± 3.02
sphingolipids	33.03 ± 20.55
Unsatured Fat Acids (UFA)	1,027.87 ± 160.462
total Diacyl Glycerophosholipids	158.48 ± 16.78
total Diacyl Glycerophosholipids (Ether)	31.57 ± 4.05
triacylglycerols	242.44 ± 28.76
total Glycerophosholipids	884.26 ± 18.13
phosphatidylcholine and sphingomyelin	1,162.56 ± 159.74
phosphatidylethanolamine	96.90 ± 14.70
poly (P)-UFA	459.87 ± 67.31
Linoleic Acid	59.13 ± 13.26
a-methylene in FA	1,004.47 ± 181.40
Mono (M)-UFA	1,037.20 ± 160.62
β-methylene in FA	2,887.70 ± 449.10
methylene in FA	20,953.50 ± 1,318.29
ωCH3 methyl in fat acids (FA)	13,341.87 ± 363.40
Total cholesterol	279.71 ± 34.69
MUFA/PUFA	2.25 ± 0.02

### 3.2 Changes in the ganglioside pattern of DPSCs during multilineage differentiation by HPTLC

**FIGURE 2:**
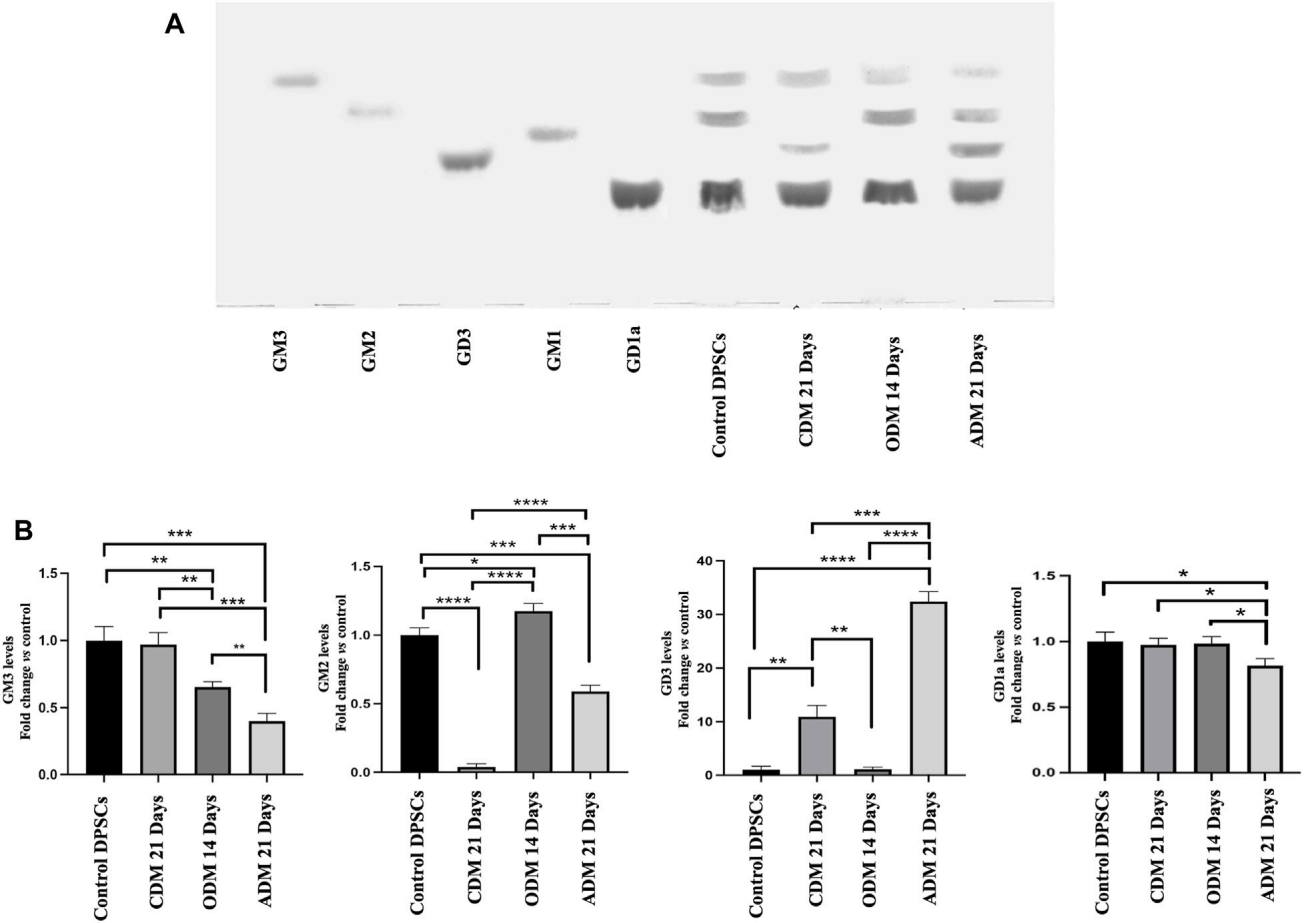
HPTLC analysis of DPSCs during multilineage differentiation. **(A)** DPSCs untreated or treated with CDM, ODM, and ADM as indicate in Table 1 were subjected to ganglioside extraction according to Svennerholm and Fredman as reported in the Materials and Methods section. Pure Standards GM3, GM2, GD3, GM1, and GD1a were used. **(B)** Densitometric analysis is shown. Results represent the mean ± SD from 3 independent experiments. *p* values for all graphs were generated using Student’s t-test as indicated in the figure *p < 0.05, **p < 0.005 ***p < 0.001, ****p < 0.0001.

In a previous work, we showed that after neuronal differentiation of DPSCs it is possible to observe a switch of gangliosides pattern (Mattei et al., 2015; Santilli et al., 2022). Therefore, we analyzed the gangliosides pattern by HPTLC during multilineage differentiation. DPSCs untreated or stimulated with Osteogenic Differentiation Medium (ODM), Adipogenic Differentiation Medium (ADM) and Chondrogenic Differentiation Medium (CDM) as described above (Table 1) were subjected to ganglioside extraction and HPTLC analysis. In Figure 2A we confirmed the basal gangliosides pattern of DPSCs (mainly GM3, GM2, and GD1a). After osteogenic differentiation process, we observed a reduction of GM3 and an increase of GM2 while, in chondrogenic differentiation we observed a reduction of GM2 and a slight increase of GD3. During adipogenic differentiation we showed the presence of GD3 and the reduction of GM3, GM2 and GD1a (Figures 2A, B).

### 3.3 Lipid rafts regulates the signal transduction pathways triggered by specific media during multilineage differentiation process of DPSCs

**FIGURE 3:**
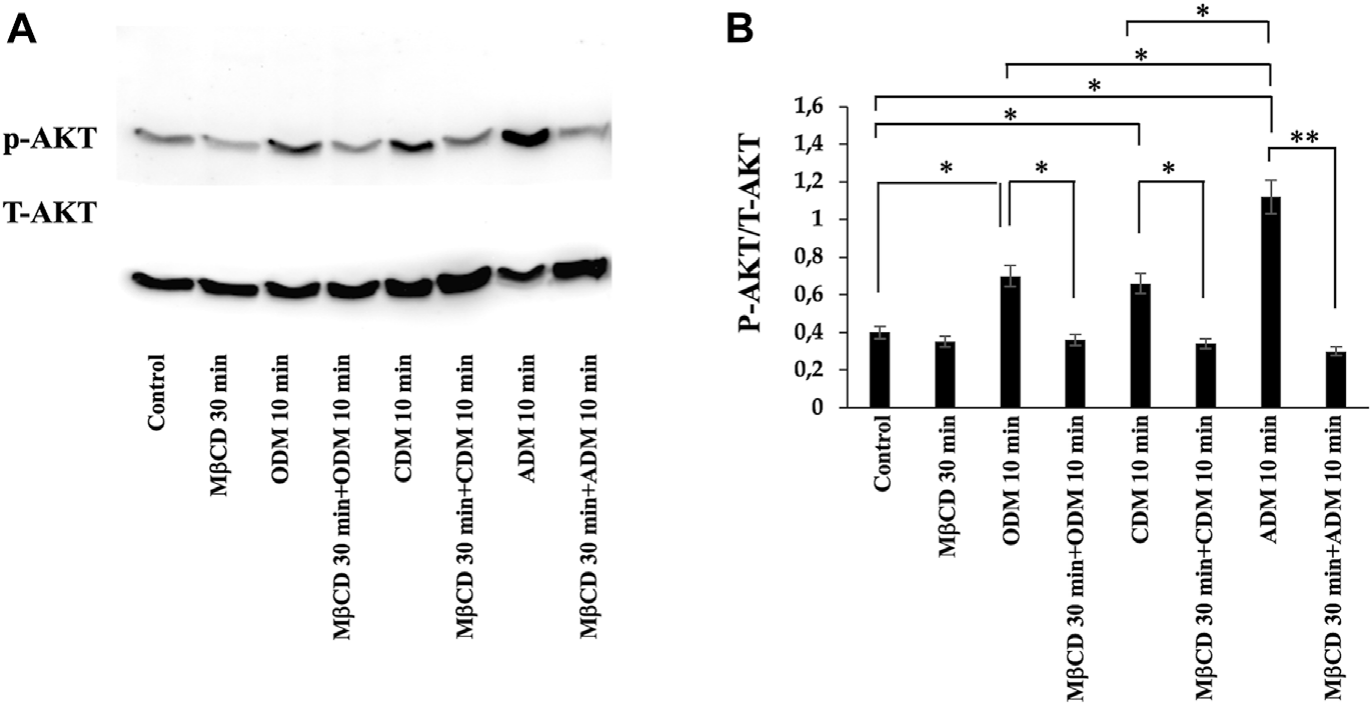
AKT phosphorylation was evaluated by Western blot analysis of DPSCs during multilineage differentiation. **(A)** DPSCs pretreated with MβCD were stimulated with ODM, ADM and CDM for 10 min and phosphorylation of AKT was evaluated by Western blot analysis using anti-phospho-AKT pAb and anti-AKT. **(B)** Densitometric analysis is shown. Results represent the mean ± SD from three independent experiments. *p* values were generated using Student’s t-test as indicated in the figure *p < 0.05, **p < 0.005.

**FIGURE 4:**
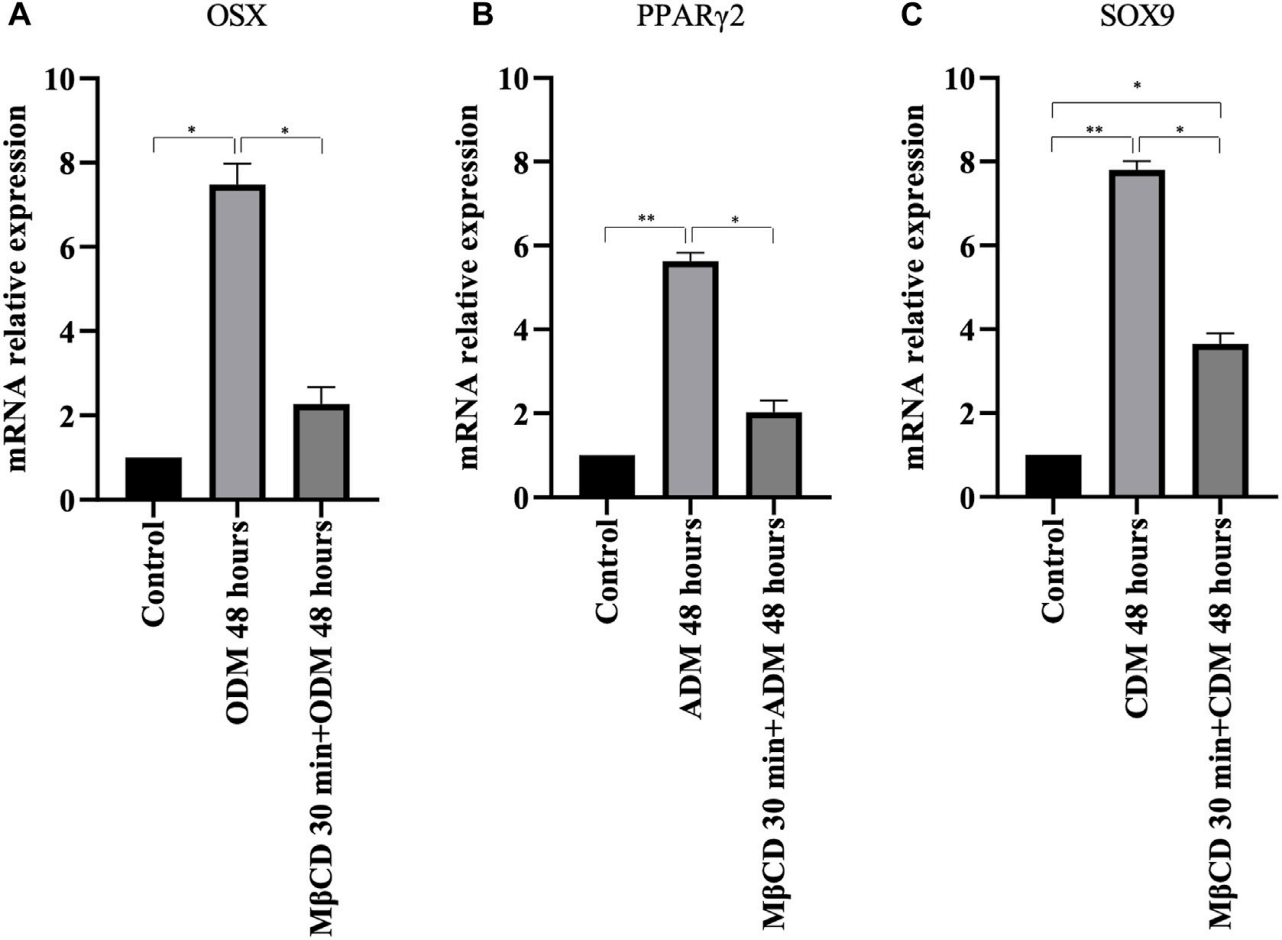
Real-time PCR. **(A)** OSX mRNA relative expression level in undifferentiated cells and cells cultured for 48 h with osteogenic differentiation medium **(B)** PPARγ2 mRNA relative expression level in undifferentiated cells and cells cultured for 48 h with adipogenic differentiation medium **(C)** SOX9 mRNA relative expression level in undifferentiated cells and cells cultured for 48 h with chondrogenic differentiation medium. *p* values were generated using Student’s t-test as indicated in the figure *p < 0.05, **p < 0.005.

To verify the possible role of lipid rafts in the signal transduction pathway involved in DPSCs multilineage differentiation, we analyzed whether MβCD may be able to regulate the activation of AKT signaling pathways. DPSCs, either untreated or treated with MβCD for 30 min at 37°C, were stimulated with specific media for multilineage differentiation process as specified above (Table 1) for 10 min at 37°C. Western blot analysis showed an increase of AKT phosphorylation levels in our DPSCs samples induced vs. multilineage differentiation, which was significantly prevented by pretreatment with MβCD (Figures 3A, B). Lipid rafts regulate the inhibition of transcription factor during multilineage differentiation process of DPSCs by Real-Time PCR. In Figure 4 we showed the increase of OSX, PPARγ2, and SOX9 mRNA expression levels during the multilineage differentiation process of DPSCs, stimulated respectively with ODM, ADM and CDM for 48 h (Table 1). Pretreatment with lipid raft-affecting agents, such as MβCD, can revert the increase of OSX, PPARγ2, and SOX9 mRNA (Figures 4A–C).

### 3.4 Role of lipid rafts during multilineage differentiation of DPSCs

**FIGURE 5:**
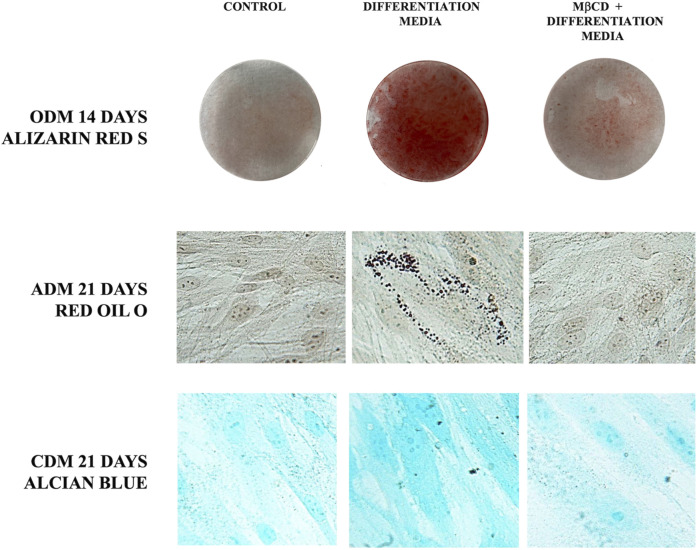
Role of lipid rafts during multilineage differentiation process. DPSCs pretreated with MβCD and then stimulated with ODM, ADM, and CDM (Table 1) were stained with Alizarin red S, Red Oil O, and Alcian blue, the typical stains used to detect osteogenic, adipogenic, and chondrogenic differentiation, respectively.

Since gangliosides have been hypothesized to be related with differentiation of DPSCs (Mattei et al., 2015; Santilli et al., 2022), we decided to analyze their role as lipid rafts components during multilineage differentiation. Thus, we pre-treated DPSCs with MβCD before inducing the differentiation process of DPSCs, as described above. Alizarin Red S staining clearly showed that pre-incubation with MβCD significantly inhibited osteoblast differentiation of DPSC cells, induced by ODM, as revealed by the inhibition of the Ca^++^ deposits formation (Figure 5). At the same time, we showed that pretreatment with MβCD, also prevented chondrogenic differentiation of DPSCs, as revealed by Alcian Blue staining specific for proteoglycans synthesis evaluation in chondrocytes (Figure 5). Finally, we analyzed the role of lipid rafts during adipogenic differentiation with the same compounds. Again, pretreatment with MβCD prevented adipogenic differentiation of DPSCs, as revealed by Oil red O used for lipid droplet staining (Figure 5).

## 4 Discussion

In the present study, we analyzed the basal lipidome and the role of lipid rafts in multilineage differentiation of DPSCs demonstrating a key role of lipid rafts during the differentiation process. The scientific literature has shown that these cells were able to differentiate into different cell types as neurons, osteoblasts, chondroblasts and adipoblasts (Geng et al., 2017; Han et al., 2019; Vidoni et al., 2019; Rafiee et al., 2020; Trejo-Iriarte et al., 2021; Guo et al., 2022). DPSCs present lipid rafts on the plasma membrane and gangliosides represent the major constituents of these plasma membrane microdomains (Ryu et al., 2009; Martellucci et al., 2018) with a critical role in stem cell differentiation (Mattei et al., 2015; Hohenwallner et al., 2022; Santilli et al., 2022). The analysis by 1H-NMR spectroscopy showed that monounsaturated fatty acid (MUFAs) were the most representative species as compared to polyunsatured fatty Acids (PUFAs) in the total pool of acyl chains. These basal composition of MUFA/PUFA ratio suggested a tight FA homeostasis for fine-tuning of membrane fluidity and permeability properties, as well as for receptor and channel functioning in DPSC cells. GGs have been debated as stem cell and lineage-specific differentiation markers. GM3, GM1, and GD2 were found to be expressed in umbilical cord and bone marrow derived MSCs (Martinez et al., 2007; Freund et al., 2010). Furthermore, GGs were differentially expressed during neural (Kwak et al., 2006; Lee et al., 2007) and osteogenic differentiation (Moussavou et al., 2013; Bergante et al., 2014; Bergante et al., 2018) while, recently it has been discovered that GGs are upregulated in adipocytes compared to their human MSC progenitors (Rampler et al., 2019). Different studies have highlighted the importance of GGs during DPSCs’ multilineage differentiation although a comprehensive analysis of the expression pattern of gangliosides in native and differentiated MSCs is missing. To date, only a few studies on MSC membrane lipids have been performed (Fuchs et al., 2007; Park et al., 2010; Campos et al., 2016). Among the different types of MSCs, Kilpinen et al., studied the effect of the donor’s age and cell doublings on the glycerophospholipids (GPL) profile of human bone marrow MSC (hBMSC) (Kilpinen, et al., 2013) and Chatgilialoglu et al., investigated how the membrane fatty acid composition of MSCs derived from human fetal membranes (hFM-MSCs) is modified by the *in vitro* change culture condition process (Chatgilialoglu et al., 2017). Several authors showed that GM2 and GD1a increase during osteogenic differentiation of DPSCs (Ryu et al., 2009; Lee et al., 2010; Moussavou et al., 2013) while GM3 and GD3 increase during chondrogenic differentiation in synovium-derived mesenchymal stem cells (Ryu et al., 2009; Lee et al., 2010). Moreover, several studies reported that after neuronal differentiation of DPSCs it’s possible to observe the expression of ganglioside GD3 (Moussavou et al., 2013; Lee et al., 2014; Martellucci et al., 2018). In this context, we analyzed the difference in gangliosides pattern of DPSCs after multilineage differentiation (osteo, chondro, adipo) under specific culture condition by HPTLC. We observed a change in gangliosides pattern during multilineage differentiation. After DPSCs’ osteogenic differentiation, we observed an increase in GM2 and GD1a, while, after chondrogenic differentiation we observed an increase in GM3 and a decrease in GM2, in adipogenic differentiation we showed the presence of GD3 and a decrease of GM3-GM2 (Figures 2A, B). GGs are the main constituent of lipid rafts, that represents a platform for protein-lipid and protein-protein interactions and for cellular signaling events. Indeed, several papers report that raft perturbation and alteration of lipid raft integrity can also affect various signaling pathways, leading to cellular death and AD (Hicks et al., 2012). Indeed, Viale-Bouroncle et al., showed that the AKT signaling pathway was activated in dental follicle cells (DFCs) after the induction of the osteogenic differentiation by Bone Morphogenetic Protein 2 (BMP2) (Viale-Bouroncle et al., 2015). Moreover, Li et al., reported that chondrogenic induction of MSCs triggered c-Myc, AKT, ERK, and MEK phosphorylation and upregulated c-Myc and mTOR expression (Li L et al., 2022). With, this idea, we analyzed whether raft affecting agents can revert the AKT phosphorylation induced by multilineage differentiation of DPSCs by ODM, CDM, and ADM. These findings confirm and extend our previous work in which we showed that lipid raft agents were able to affect neuronal differentiation process in DPSCs (Mattei et al., 2015). On this basis, we evaluated the ability of MβCD to revert the expression of mRNA during the multilineage differentiation process induced by ODM, CDM, and ADM. Real-time PCR showed that MβCD can revert the mRNA expression of OSX, SOX9, and PPARγ2. Moreover, we used Alizarin Red S, Alcian Blue, and Oil red O solution to stain the DPSCs after differentiation, respectively, in osteoblasts, chondroblasts, and adipoblasts cells. We showed that pretreatment with MβCD prevented osteogenic, chondrogenic and adipogenic differentiation of DPSCs, as revealed by reduction of staining with Alizarin Red S, Alcian Blue and Oil red O. In conclusion, in this paper we observed a change in the ganglioside pattern during the multilineage differentiation processes and demonstrated that lipid rafts are essential components involved in the multilineage differentiation process. All these data highlight how the structural integrity of lipid rafts is essential in the multilineage differentiation process of DPSCs and that this mechanism is mediated by AKT phosphorylation.

## 5 Conclusion

This paper demonstrates a change in the ganglioside pattern during the multilineage differentiation processes, indicating that lipid rafts are essential components involved in the multilineage differentiation process. Moreover, the perturbation in lipid raft compositions with MβCD affected Akt signaling pathway that was activated in response to differentiation stimuli. In this concern, regulation of lipid raft pattern may play a pivotal role in committing these cells to differentiate into other cell types or self-renew. Therefore, understanding the role of protein interacting lipids in DPSCs will greatly benefit and improve regenerative medicine, specially related to tissues utilizing lipids as energy source.

## Data Availability

The raw data supporting the conclusion of this article will be made available by the authors, without undue reservation.
