# Supplementary material for: Lipid rafts mediate multilineage differentiation of human dental pulp-derived stem cells (DPSCs)
Source: Front Cell Dev Biol. 2023 Nov 9;11:1274462. doi: 10.3389/fcell.2023.1274462 (PMC10665896; doi:10.3389/fcell.2023.1274462)
Supplement: Supplementary file 1 [file Table1.docx]

**Table S1**: 1H-NMR lipid metabolites assignment (protons and chemical shift at 600 MHz) of intracellular lipidome identified in organic fraction of hDSPC cells. Abbreviations: FAs, fatty acids; GPL, glycerophospholipids; LA, Linoleic acid; MUFAs, monounsaturated fatty acids; PC, phosphatidylcholine; PE, phosphatidylethanolamine; PLS, plasmalogens; PUFAs, polyunsaturated fatty acids; SL, sphingolipids; SM, sphingomyelin; TAGs, triacylglycerides; UFAs, unsaturated fatty acids. In red bold the protons responsible for selected signals for lipid quantification.

| **Metabolites** | **^1^H NMR peak assignment** | **Chemical shift (ppm)** | **Quantification of Lipid metabolites** |
| --- | --- | --- | --- |
| ***Cholesterol molecule*** | C_18_***H***_3_ | **0.70** | **Total cholesterol**, |
| ***Glycerophospholipids (GPL) and sphingolipids (SL)*** | 1-C**H_2_**/3-C**H_2_** of glycerol | **4.18** | **Total TAG** |
|  | C_1_***H***^d^and C_3_***H***^d^of glycerol backbone of DAGPLs | **4.40** | **Total Diacyl glycerophospholipids (DAGPLs)** |
|  | C_2_***H*** of glycerol backbone in ether glycerophospholipids | **5.17** | **Ether GPLs** |
|  | C_2_***H*** of glycerol backbone in total DAGPLs | **5.24** | **Total Diacyl glycerophospholipids (DAGPLs)** |
|  | -CH_2_-CH_2_-N^+^(C***H***_3_)_3_(choline) | **3.20** | **Total choline-containing PLs (PC, SM)** |
|  | -CH_2_-C***H***_2_-NH_3_^+^(ethanolamine) | **3.10** | **PE** |
|  | -OC***H***=CHCH_2_ | **5.92** | **plasmalogens (ether GPLs)** |
| **Fatty acids (FA)** | -(C***H***_2_)_n_- (methylene) in fatty acyl chains | **1.30** | **Total FA** |
|  | -CO-CH_2_-C***H***_2_- (β-methylene) in the fatty acyl chains | **1.60** | **Total FA** |
|  | -C***H***_2_-CH =(allylic) in fatty acyl chains | **2.01** | **MUFA** |
|  | -CO-C***H***_2_ (α-methylene) in the fatty acyl chains | **2.33** | **Total FA** |
|  | -CH=CH-C***H***_2_-CH=CH- of linoleic acid | **2.78** | **LA (18:2 ω-6)** |
|  | -(CH=CH-C***H***_2_-CH=CH)n, n>1 in the fatty acyl chains | **2.84** | **PUFA** |
|  | Terminal-C***H***_3_ in the fatty acyl chains | **0.89** | **Total FA** |
|  | -C***H***=C***H***- in the fatty acyl chains | **5.36** | **UFA** |
